# Functionalization of Microfiltration Media Towards Catalytic Hydrogenation of Selected Halo-Organics from Water

**DOI:** 10.3390/nano16010014

**Published:** 2025-12-22

**Authors:** Subrajit Bosu, Samuel S. Thompson, Doo Young Kim, Noah D. Meeks, Dibakar Bhattacharyya

**Affiliations:** 1 Department of Chemical and Materials Engineering, University of Kentucky, Lexington, KY 40506, USA; subrajit.bosu@uky.edu (S.B.); sam.thompson@uky.edu (S.S.T.); 2 Department of Chemistry, University of Kentucky, Lexington, KY 40506, USA; dooyoung.kim@uky.edu; 3 Southern Company Services, Inc., Birmingham, AL 35203, USA; ndmeeks@southernco.com

**Keywords:** wastewater, membrane, fiber, hydrogenation, hydrogen utilization, nanoparticles, catalyst

## Abstract

Contaminated water detoxification remains difficult due to the presence of persistent halo-organic contaminants, such as perfluorooctanoic acid (PFOA) and chlorophenols, which are chemically stable and resist conventional purification methods. Functionalized membrane-based separation and decontamination have garnered immense attention in recent years. Commercially available microfiltration membrane (PVDF) and polymeric non-woven fiber filters (glass and composite) are functionalized with poly(methacrylic acid) (PMAA) that shows outstanding pH-responsive performance and tunable water permeability under ambient conditions perfect for environmental applications. Polymer loading based on weight gain measurements on PMAA–microglass composite fibers (137%) and microglass fibers (116%) confirmed their extent of functionalization, which was significantly greater than that of PVDF (25%) due to its widely effective pore diameter. Presence of chemically active hydrogel within PVDF matrix was validated by FTIR (hydroxyl/carbonyl) stretch peak, substantial decrease in contact angle (68.8° ± 0.5° to 30.8° ± 1.9°), and decrease in pure water flux from 509 to 148 LMH/bar. Nanoparticles are generated both in solution and within PVDF using simple redox reactions. This strategy is extended to PVDF-PMAA membranes, which are loaded with Fe/Pd nanoparticles for catalytic conversion of 4-chlorophenol and PFOA, forming Fe/Pd-PVDF-PMAA systems. A total of 0.25 mg/L Fe/Pd nanoparticles synthesized in solution displayed alloy-type structures and demonstrated a strong catalytic performance, achieving complete hydrogenation of 4-chlorophenol to phenol and 67% hydrogenation of PFOA to its reduced form at 22–23 °C with ultrapure hydrogen gas supply at pH 5.7. These results underscore the potential of hybrid polymer–nanoparticle systems as a novel remediation strategy, integrating tunable separation with catalytic degradation to overcome the limitations of conventional water treatment methods.

## 1. Introduction

The scarcity of freshwater supply has resulted in a growing concern about the contamination of urban and rural water sources where there is an increasing discharge of pathogenic medicinal products, cosmetics, phosphate-rich detergents, petrochemicals, heavy metals, nanoplastics, agrochemical waste, landfill leachate, and anthropogenic chemicals, such as poly or perfluoroalkyl substances (PFASs). They are some of the prevalent contaminants in our environment which exhibit distinct physicochemical properties based on partial or fully fluorinated carbon skeleton [1,2,3,4]. Since the 1950s, nearly 15,000 PFAS-containing industrial products have become deeply integrated into our everyday life [5,6,7], and health researchers have linked these chemicals to a wide range of adverse effects, including neurotoxicity, endocrine imbalances, and reproductive impairment—impacts that also extend to wildlife and marine organisms [8]. PFOA (perfluorooctanoic acid) and PFOS (perfluoro octane-sulfonic acid) are called legacy PFAS as they have been completely phased out of production in many developed countries with US EPA recently setting drinking water PFOA and PFOS levels at 4 ppt (ng/L) following risk and toxicity analyses over the years [9]. Adsorption [10], nanofiltration [11], advanced reduction/oxidation processes [12], and bacterial decomposition [13] coupled with the development of photocatalytic systems [14] are well-established for the removal or destruction of long-chain PFOA molecules. However, the formation of shorter chain PFAS that are more water soluble plus mobile, use of harsh reaction conditions, slow reaction kinetics, and the cost of secondary pollution, which is expensive, mitigate the scale-up potential for easy and sustainable development of water treatment technology [15,16]. Although membrane-based purification is well-established, the evolving composition of wastewater necessitates continual improvements to performance, spatial efficiency, energy consumption, permeate purity, and operational simplicity [17,18]. A polymeric membrane is commonly used in separation [19] and purification [20] because of its highly adaptable properties since its pore size distribution can be tailored to meet a variety of applications depending on the casting conditions. Despite these advantages, polymeric membranes are limited in their lifespan by their high hydrophobicity and susceptibility to fouling. They are also very sensitive to bleach washing [21]. To overcome these limitations, nanostructured materials and functional groups are incorporated onto membranes to impart unique morphological, mechanical, and chemical properties [22]. Recent research into functionalized membranes and fiber media with their porous support, as well as various responsive polymers, has enabled precise control of pore architecture, permeability, and selectivity [23,24,25]. PVDFs are noteworthy because of their ease of functionalization and stable thermal, chemical, and mechanical properties. As one of the most versatile membranes available, PVDFs have outstanding physical, chemical, and thermal properties. It is common for water streams to exhibit a wide range of pH conditions during remediation operations. Furthermore, membranes functionalized with pH-responsive polymers have the advantage of being able to modulate their permeability, swelling, and surface charge using protonation–deprotonation [26,27]. When weak acidic polymers, such as polyacrylic acid, polymethacrylic acid, or polyvinyl pyridine, are combined with suitable crosslinkers and initiators, homogenous hydrogel networks are created with enhanced selectivity and adaptive properties [28,29]. Furthermore, metal encapsulation within pores [30], fabrication of inorganic nonmetals [31], and addition of enzymes to membrane matrixes have leveraged separation performance [32]. Chemical reduction is proven to be very effective using precious metal such as Pt, Pd, Rh, and Ru, which utilizes molecular hydrogen to selectively convert halates/halorganics into halides/dehalogenated compounds and nitrates/nitrites into nitrogen/ammonia by H* radical substitution with a halogen atom in a carbon chain [33,34]. The earth has abundant Cu, Fe, Co, Zn, and Ni compared to expensive Pd, and these elements can activate H2 to remove pollutants or participate directly in pollutant reduction by donating electrons [35,36]. However, their bulk performance in aqueous matrix under ambient conditions is relatively poor [37]. This is due to their low level of inherent activity where Pd outstands other metals due to its chemical transition properties. Pd’s unfilled electron orbital easily binds reactants to its surface. The zero-valent Palladium (Pd^0^) can be easily oxidized into Pd(I) or Pd (II) due to its good nucleophilicity. As a result of its ability to nucleophilically combine with organic groups, Pd0 has been extensively studied for its use in synthesis and exceptional hydrogenation [38]. Due to their electron density acceptance, air stability, and capacity to be dissolved in a variety of organic solvents, Pd(I) and Pd (II) are good electrophiles. Notably, the Pd2+/Pd redox couple shows a higher standard reduction potential in comparison with other transition metals [39]. When combined with other metals, palladium-based systems demonstrate significantly stronger and more efficient detoxification performance [40]. Although catalytic hydrogenation of halo-organic compounds using Pd has been widely studied, only a limited number of investigations have focused on PFOA, particularly under mild or ambient conditions.

**Figure 1:**
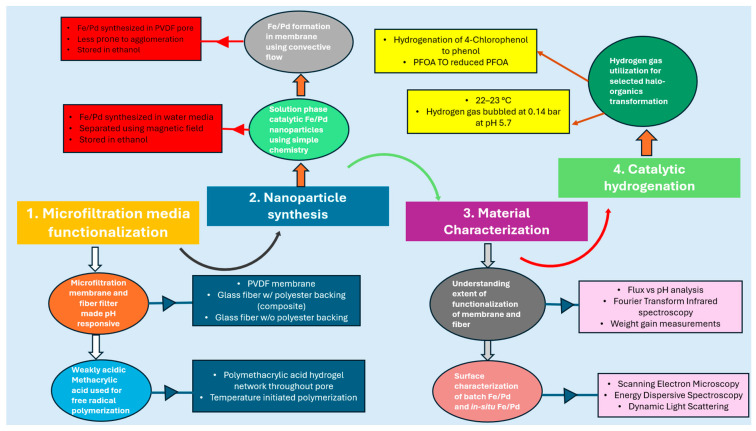
Illustrative schematic of experimental workflow in this study.

To fill in the key gaps identified in the existing research, the study aims to incorporate functional domain into commercially available microfiltration membrane and filter fiber media illustrated in Figure 1. In support of this approach, the study sets forth the following experimental objectives: (1) functionalize microfiltration membrane and polymeric non-woven fiber filters using pH-responsive methacrylic acid exhibiting tunable water flux, (2) synthesis of solution phase catalytic Fe/Pd nanoparticles and incorporation of Fe/Pd only within membrane pores via convective flow redox chemistry, (3) characterize membrane and fiber media comprehensively through evaluation of membrane functionalization, and detailed physicochemical characterization of both solution-phase nanoparticles and in situ synthesized nanoparticles, (4) evaluate hydrogenation performance by conducting batch and convective flow studies of 4-chlorophenol (4-CP) and PFOA to assess catalytic effectiveness of Fe/Pd-loaded microfiltration membrane (PVDF-650) at 22–23 °C to transform toxic substances into mildly toxic substances or compounds with lower toxicity potential. This transformation uses reductive hydrogenation pathways compared to advanced oxidation which usually requires harsh reaction conditions.

## 2. Materials and Methods

### 2.1. Materials

All chemicals used for membrane functionalization and associated laboratory experiments were of analytical grade and applied without further purification. Commercial PVDF 650 microfiltration membranes (thickness 178 µm) were supplied by Solecta Inc. (Oceanside, CA, USA). Polymeric non-woven fiber filters such as bare microglass (thickness 647 µm) and microglass with polyester backing also called microglass composite fibers (thickness 830 µm) were obtained from Atmus filtration Inc. (Cookeville, TN, USA). Iron (III) chloride hexahydrate (98%) and iron (II) chloride tetrahydrate (98%) were obtained from Alfa Aesar (Haverhill, MA, USA). ASTM Type 1–2 water (ACS reagent grade, RICCA), ammonium persulfate (APS), and N,N′-methylenebisacrylamide (MBA, 99+%, Thermo Scientific, Waltham, MA, USA) were employed as received. Sodium hydroxide (0.1 N) and hydrochloric acid (0.1 N) were procured from Fisher Scientific (Waltham, MA, USA), while sodium chloride was sourced from ACS Organics (Berkeley Heights, NJ, USA). Deoxygenated water was prepared by purging nitrogen into deionized ultra-filtered (DIUF) water for 30 min. Potassium tetrachloropalladate was purchased from Strem Chemicals (Newburyport, MA, USA). 4-Chlorophenol (4-CP) and Omnipure ethanol were used for nanoparticle storage and treated for membrane preservation; Perfluorooctanoic acid (PFOA, 95%) and Pd on aluminum (1 wt% Pd loading) were purchased from Sigma Aldrich (St. Louis, MO, USA). Sodium borohydride (99% reagent grade) was obtained from Acros Organics (Waltham, MA, USA).

### 2.2. Functionalization of PVDF and Fiber Filters

**Figure 2:**
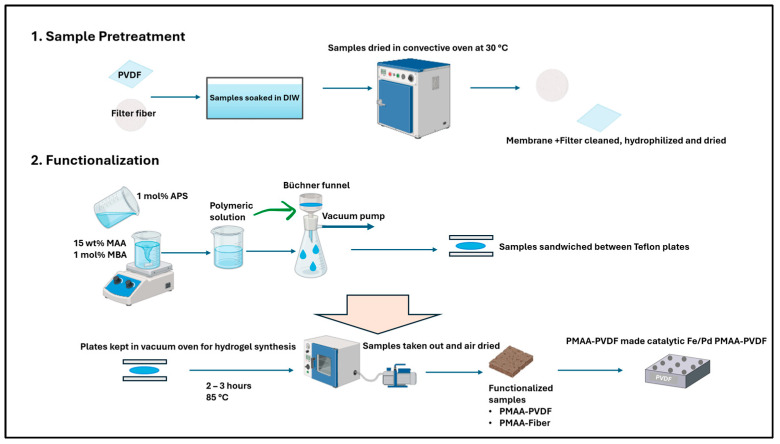
Synthesis of functionalized membranes and fiber media, created using Biorender.

Functionalization was carried out on PVDF-650 microfiltration membranes and polymeric non-woven fiber filters as illustrated in Figure 2. The fiber media comprised two variants: bare microglass and microglass supported with a polyester backing (also referred to as microglass composite throughout the study). Commercially available PVDF-650 microfiltration membranes are soaked in deionized water to clean and hydrophilize the pores at pH 5.7 for 4.5 h at ambient conditions, and later dried in a convective oven at 30 °C (fan 40%) for 3 h. The membranes are then functionalized with polymer solution containing 15 wt% MAA, 1 mol% APS, and MBA crosslinker, passed through the Büchner funnel using vacuum filtration, and later cooked in the vacuum oven for almost 2 h. at 85 °C, sandwiched between Teflon plates. Deoxygenated water is used throughout the system to eliminate dissolved O_2_ that acts as a scavenger for polymerization. The membrane is taken out and air dried for 1 h before measuring its functionalized weight. A similar approach is used for microglass and microglass composite fiber filters, except the fiber filters are kept in the oven at the same temperature for 2:30–3 h as it had a substantial weight gain compared to the membrane; this was followed by air drying for one more hour to ensure all moisture is lost for dry mass measurement. In our permeability studies, we used stainless steel Sterlitech (Auburn, WA, USA) part number 4750 dead-end filtration cells with a 14.6 cm^2^ membrane active area and solvent-resistant stirred cell from Millipore sigma (Burlington, MA, USA) part number XFUF07601 with a 40 cm^2^ filtration area. An overview of the microfiltration media used for this study is shown in Table 1.

### 2.3. Synthesis of Fe/Pd Nanoparticles in Pore of PVDF

**Figure 3:**
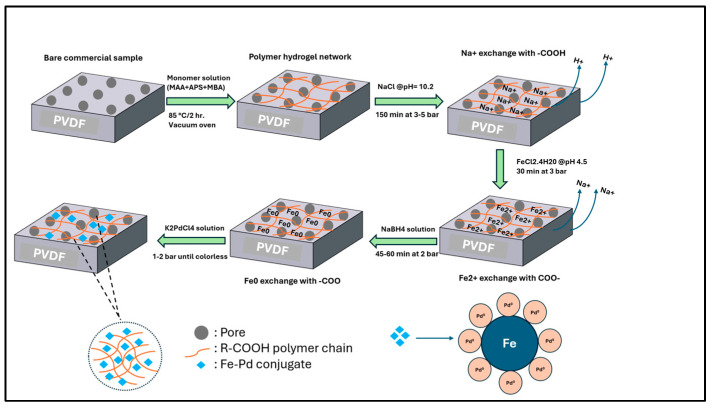
Schematic of the synthesis procedure of Fe/Pd PMAA-PVDF through convective flow at 22–23 °C.

The Fe/Pd loading process onto the membrane domain involves several sequential steps, as shown in Figure 3 below. First, 300 mL of a 68 mM sodium chloride (NaCl) solution (prepared by dissolving 1.19 g of NaCl) is adjusted to pH 10.6 and passed through the PMAA-PVDF650 at a pressure of 3–7 bar for 150 min. This step facilitates Na^+^ ion coordination with the polymer and proton release, evidenced by a decrease in permeate pH to 3.8, which promotes further ion-exchange with Fe^2+^. Next, 200 mL of a 3.57 mM ferrous chloride tetrahydrate (FeCl_2_·4H_2_O) solution (containing 0.040 g of Fe from 0.142 g of salt) is passed through the membrane at 1–2 bar and pH 4.2 for 30 min to displace Na^+^ and Cl^−^ ions. The immobilized Fe^2+^ ions are then reduced to zero-valent iron (Fe^0^) by introducing 300 mL of a 26 mM sodium borohydride (NaBH_4_) solution at 3–5 bar, continuing until black coloration is observed, indicating Fe^0^ formation. Finally, 200 mL of a mixed ethanol–water solution (9:1 *v*/*v*) containing 153 µM K_2_PdCl_4_ (equivalent to 1 wt% Pd relative to Fe, or 9.99 mg of salt) is passed through the membrane to deposit palladium. The completion of Pd loading is confirmed by the color change in the solution from light brown to clear.

### 2.4. Synthesis of Fe/Pd Nanoparticles in Solution Phase

**Figure 4:**
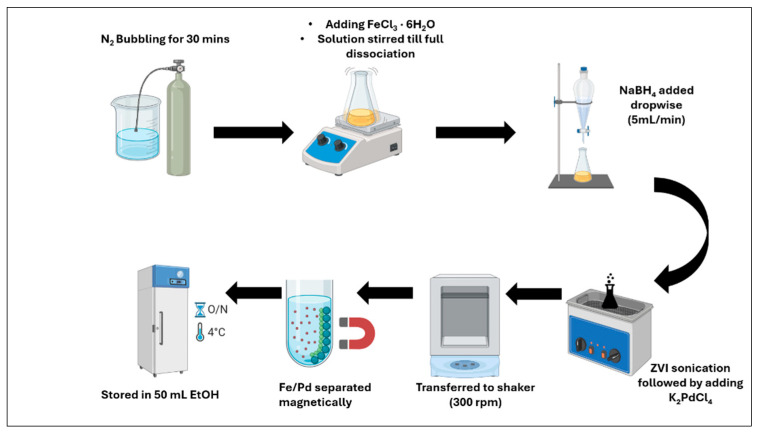
Detailed experimental workflow of solution phase catalytic Fe/Pd used for 4-Chorophenol and PFOA hydrogenation at 22–23 °C, created using Biorender.

Prepare a 100 mL solvent mixture composed of 40% deionized water and 60% ethanol and purge it with nitrogen gas for 30 min to remove dissolved oxygen. Dissolve 1 g of ferric chloride hexahydrate in the deoxygenated solution, causing the color to shift from clear to brown. Separately, dissolve 0.5 g of sodium borohydride in 100 mL of freshly deoxygenated deionized water. Add this NaBH_4_ solution dropwise (approximately 5 mL/min) to the Fe^3+^ solution under continuous stirring at 100 rpm for 60 min, until the solution changes from brown to black, indicating the formation of the zero-valent iron (ZVI). Use cold packs around the reaction vessel to maintain a safe temperature. Collect the ZVI particles magnetically and wash them two to three times with pure ethanol. Seal the resulting suspension and sonicate for 5–10 min to maximize surface area exposure for palladium deposition. Separately, dissolve 0.05 g of potassium tetrachloropalladate (II) (K_2_PdCl_4_) in 50 mL of ethanol. Combine this Pd solution with the ZVI dispersion and stir at 300 rpm for 45 min. Recover the synthesized Fe/Pd bimetallic nanoparticles using a strong magnet, rinse with ethanol three times to remove unwanted ions, and store the solid particles in 50 mL ethanol with minimal headspace to reduce oxidation of ZVI illustrated in Figure 4 below.

### 2.5. Material Characterization

#### 2.5.1. Membrane and Filter Topography Analysis

The surface morphology of the bare fibers and microfiltration membrane (PVDF 650), as well as the PMAA-PVDF 650 and Fe/Pd PMAA PVDF 650 membranes, is studied using a scanning electron microscope (SEM Quanta, Hitachi S-4300, Hitachi, Tokyo, Japan). Bare and PMAA-PVDF650 membrane samples were dried to ensure no moisture before being sputter-coated with platinum to make the surface charged and reduce electron accumulation to overcome the apparent brightness of images. The Leica EM ACE600 sputtering system (Leica Microsystems, Wetzlar, Germany) was used to deposit 5.63 nm Pt at 0.12 nm/s at 31 °C.

#### 2.5.2. Weight Gain Measurements

The average weight gain post-functionalization of the bare non-woven fibers (with and without polyester backing) and PVDF 650 is measured based on the weight of dry bare samples (Wd) and PMAA-loaded functionalized samples (Wf) using the following equation:

(1) $$\mathrm{Weight}\;\mathrm{gain}\;\%=\frac{Wf\left(g\right)-Wd(g)}{Wd(g)}\times 100\%$$

Following 24 h drying in a convective oven at 35 °C (fan 40%), all functionalized samples are weighed. This ensures that all moisture is lost, and any weight gain is solely due to the incorporation of PMMA.

#### 2.5.3. PMAA Incorporation onto Membranes

The successful synthesis of the hydrogel was visually indicated by the appearance of a white, cloudy, jelly-like material in a glass vial. Elemental composition analysis was carried out using Fourier transform infrared spectroscopy (Nicolet iS50 FT-IR, Thermo Scientific, Waltham, MA, USA) to qualitatively confirm functionalization with PMAA. FT-IR spectra were collected in the range of 500–4000 cm^−1^, with an average of 32 scans, employing a potassium bromide crystal detector. Surface wettability was assessed through contact angle measurements, using 10 µL droplets of deionized water (pH~5.7) on a Krüss DSA100S drop shape analyzer (Matthews, NC, USA).

#### 2.5.4. Characterization of Fe/Pd Catalytic Nanoparticles in Solution Phase

To evaluate the effectiveness of Pd deposition via zero-valent iron reduction, Fe/Pd nanoparticles were synthesized in solution phase and analyzed using X-ray diffraction (Siemens D500; Siemens, Munich, Germany) with Cu Kα radiation (λ = 1.5418 Å). The XRD was operated at 40 kV and 30 mA. Prior to analysis, the ethanol solvent evaporated, and the dried powders were mounted on glass microscope slides and compressed to create a uniform surface. Diffraction spectra were recorded over a 2θ range of 10–100°, using a step size of 0.05° and a dwell time of 1 s per step.

With dynamic light scattering (DLS), the size and distribution of fresh Fe/Pd particles were evaluated. A total of 3 mL of samples was placed in quartz/disposable cuvettes, preventing air bubbles from forming. Kimwipes were used to clean the exterior of the cuvette to remove dust and fingerprints. The samples were tested in triplicate with an Anton Paar DLS particle analyzer at 22 °C. Each measurement was preceded by a quick vortexing of the samples to avoid agglomeration of the particles. The Stokes–Einstein equation was used to calculate particle sizes, and the results were reported as mean hydrodynamic diameters. In order to determine the uniformity of the particle size distribution, the polydispersity index (PDI) was used.

#### 2.5.5. Morphology and Composition of Fe/Pd Nanoparticles Within the Membrane

The morphology and distribution of Fe/Pd nanoparticles on the surface were investigated, followed by elemental analysis of selected regions using a Quanta FEG 250 E-SEM equipped with EDX (Oxford Instruments, Oxford, UK). While individual nanoparticles could not be distinctly resolved in the EDX elemental maps, agglomerated clusters were clearly identifiable. Elemental composition was therefore determined for these aggregated zones, focusing solely on Fe and Pd. Other detected elements—such as C, H, F, and O originating from the membrane, Pt from the conductive coating, and Cu from the sample holder—were excluded to ensure accurate analysis of Fe and Pd content [41].

### 2.6. PFOA and 4-Chlorophenol (4-CP) Hydrogenation

**Figure 5:**
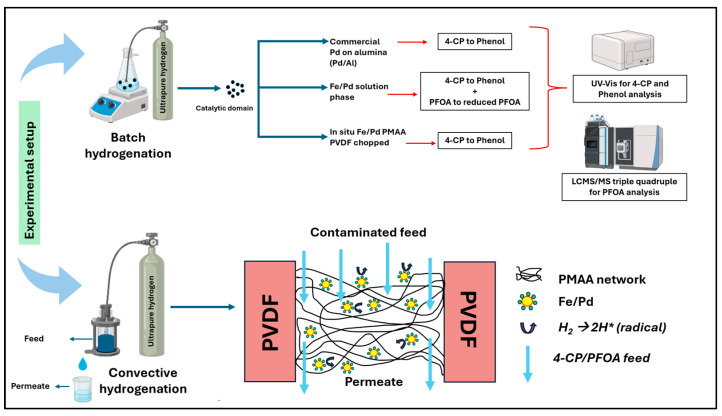
Detailed experimental workflow for 4-Chorophenol and PFOA hydrogenation at 22–23 °C, created using Biorender.

A series of preliminary batch and convective degradation experiments with 4-chlorophenol (4-CP) as a model contaminant for its ease of analysis and phenol as the transformation product was carried out to evaluate the catalytic efficiency of the developed system as shown in Figure 5. PFOA was used as the primary pollutant to check whether the developed catalytic domain had potential for degradation. All experiments were performed at ambient temperature and there was no use of toxic/harsh solvents. Hydrogen gas supplies directly to reaction chambers to form bubbles using a sparger and overcomes the limitations of H_2_ supply for C-F bond cleavage, rather than the hydrogen produced from ZVI corrosion employed by our group in previous PCBs degradation experiments for the enhanced degradation of PCBs. UV–vis analyses were performed with a UV-6300PC spectrophotometer (VWR International, Leuven, Belgium), using 278 nm to quantify the characteristic peak of 4-chlorophenol and 268 nm for phenol detection. All 4-CP samples were then diluted to reach peak absorbance values under one, thus still following the Beer–Lambert Law. pH was also measured and adjusted (using 0.1 N NaOH/HCl) if necessary to ensure all UV-vis measurements for 4-CP were taken at the same pH. Targeted PFAS (specifically PFOA) analysis was performed using liquid chromatography–triple quadruple mass spectroscopy (LC/MS/MS Agilent 6470 LC/TQ, Santa Clara, CA, USA) with a PFC-free kit. Chromatography used LCMS-grade water with 10 mMol ammonium acetate and LCMS-grade methanol as its aqueous and organic phases, respectively. For mass spectroscopy of PFOA and PFBA, EPA method 533 was followed, with some exceptions. The sheath gas temperature was set to 350 °C, the gas temperature was set to 230 °C, the gas flow was set to 10 L/min, the nebulizer was set to 3.1 bar, the voltage was set to 2500 V, and the polarity was negative for all compounds examined. The initial PFOA concentration in the feed was 1 mg/L, which was subsequently diluted to 100 ppb prior to injection to meet LC column requirements and to simulate wastewater treatment scenarios involving concentrated PFOA streams [42,43]

## 3. Results

This experimental section presents the functionalization, analysis, and performance testing of pH-responsive PVDF-650 membranes and fibers. All hydrogenation tests are restricted to solution phase Fe/Pd and Fe/Pd PMAA-PVDF650. Gel appearance, weight gain measurements, pH-responsive behavior, FTIR spectra, and contact angle measurements were used to prove functionalization. XRD, EDX, SEM, and DLS were used to characterize solution-phase Fe/Pd nanoparticles, while SEM and EDX were used to visualize in situ nanoparticle formation within PMAA-PVDF650 membrane pores. Model pollutants 4-CP and targeted pollutant PFOA were used to test catalytic activity in batch and convective flow modes. In the Section 4, perspectives are presented on expanding applications to other emerging contaminants.

### 3.1. Understanding the Extent of Functionalization of Microfiltration Membrane and Fiber Media

The initial confirmation for the synthesis of the hydrogel/polymer network is the gel appearance as shown in Figure S1, which is the residual monomer solution after functionalization. Based on visual examination of the gels, a soft, uniform, and slightly translucent hydrogel was formed at 1 mol% MBA crosslinker, indicating high water retention and network flexibility. Across multiple synthesis batches, gel consistency was demonstrated to be repeatable. At 2 mol% MBA concentrations, we observed opaque materials, indicating excessive crosslinking density, which prevented polymer chain mobility and reduced water absorption. This is consistent with hydrogel theory, which states that an increased crosslinker content leads to a harder, glassier gel structure because swelling ability decreases and stiffness increases. The gel texture of the two formulations differs significantly, demonstrating that crosslinker concentration greatly influences the physical characteristics of the hydrogel, which influences membrane pH sensitivity and nanoparticle loading [44].

**Figure 6:**
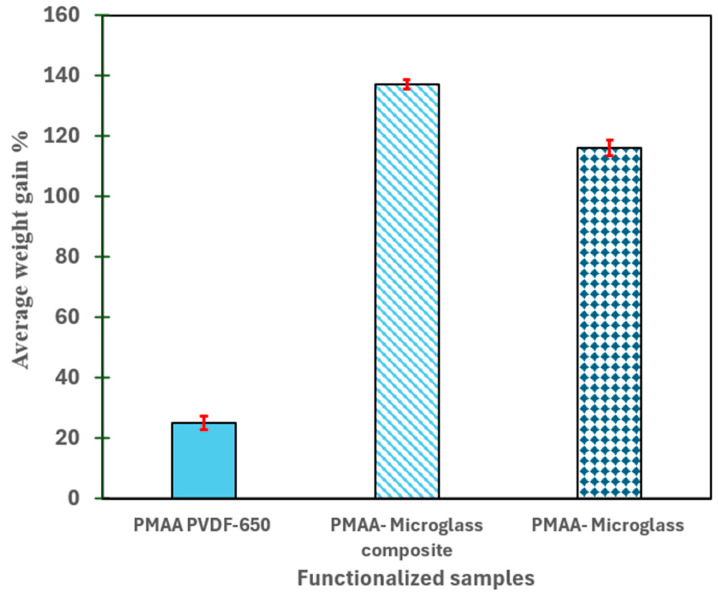
Average weight gain% of PMAA-loaded microfiltration membrane (PVDF-650), microglass composite fiber filter (with polyester backing), microglass with 15 wt% PMAA and 1 mol% APS initiator, and MBA crosslinker functionalized for 2 h at 85 °C. Error bars are shown for triplicate measurements.

The PVDF, microglass, and microglass composite experienced substantial weight gain. The polymer loading onto microglass composite fibers (137%) and pristine microglass fibers (116%) were significantly greater than that of PVDF650 (25%), as shown in Figure 6; since the surface wettability of fiber is higher, initiators are effectively absorbed, and reactive groups are abundant, such as Si-O in microglass and polar COO- functional groups in polyester repeating units. In addition to providing extended radical generation, the open fibrous structure (illustrated in Figure S2A–F) facilitates the formation of homogeneous polymer networks. The hydrophobicity and chemical inertness of PVDF prevents it from penetrating MAA units, which reduces free radical formation. This results in relatively low mass gains.

**Figure 7:**
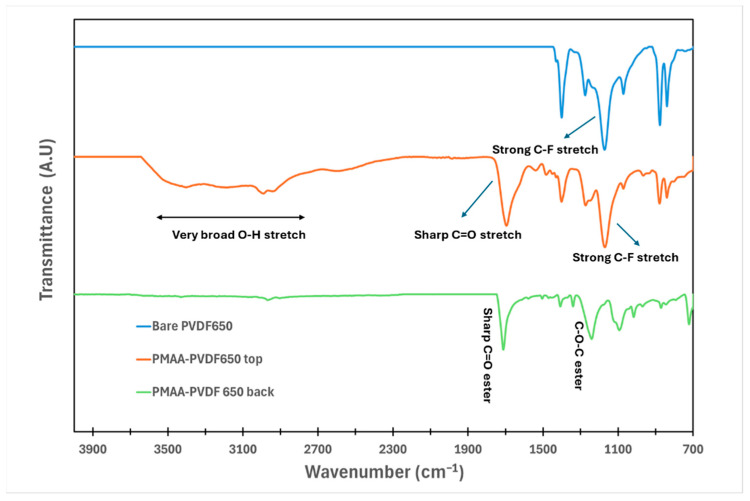
ATR-FTIR of bare and PMAA-functionalized microfiltration membrane (PVDF-650) collected in the range of 500–4000 cm^−1^ with an average of 32 scans.

ATR-FTIR analysis confirmed successful surface functionalization of PVDF-650. Background spectra were recorded for each run and subtracted to eliminate noise. As depicted in Figure 7, the characteristic CF/CF_2_ stretching band around 1166–1404 cm^−1^ present in the bare PVDF and in PMAA-PVDF was also observed in PVDF powder, consistent with our group’s earlier findings [29,45]. In the PMAA-PVDF sample, additional peaks emerged. A distinct polar C=O stretching band at 1709–1735 cm^−1^ and a very broad O–H stretching band spanning 2900–3800 cm^−1^ due to surface PMAA hydrogel network after functionalization was observed. These spectral features provide clear evidence of PMAA incorporation into the PVDF structure. Furthermore, FTIR spectra of PMAA-PVDF650 back shows sharp peaks at 1330–1345 cm^−1^ due to the C-O group and at 1670–1735 cm^−1^ due to the C=O group coming from the polyester backing in the PVDF module, which is consistent with the literature [46].

Drop in pure water permeability after functionalization proves the successful formation of hydrogel matrix within the sample. The pure water permeability of the bare PVDF-650 membrane is 509 LMH/bar, while the bare microglass and the microglass composite exhibited much higher initial permeabilities of 3392 LMH/bar and 5144 LMH/bar, respectively. After functionalization, these values dropped to 148 LMH/bar for PVDF-650, 5.6 LMH/bar for the microglass, and 120 LMH/bar for the microglass composite with all permeation experiments conducted in triplicate at pH 5.7. After in situ catalyst formation, Fe/Pd PMAA PVDF-650 shows a permeability of 20.7 LMH/bar shown in Figure S3, which is the same as Fe PMAA PVDF-650, since Pd loading had no significant flux drop.

**Figure 8:**
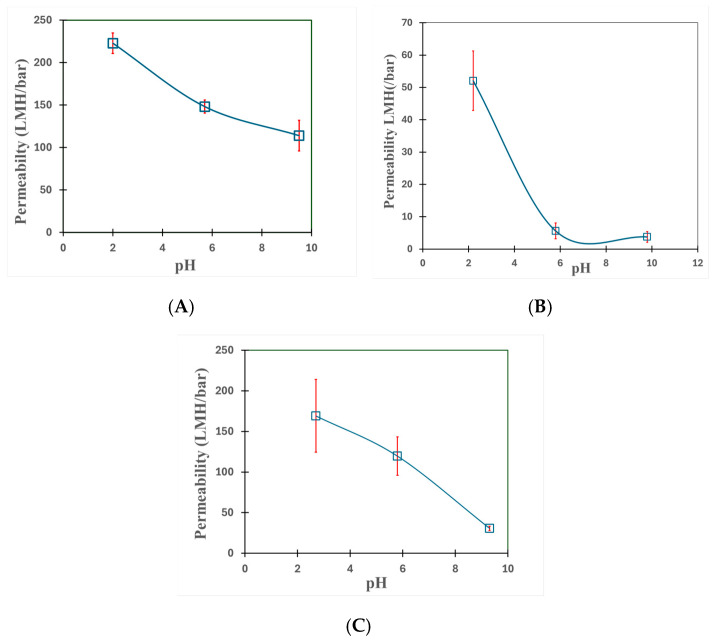
Pure water permeation experiments (applied pressure = 2–6 bar; temperature = 22–23 °C; pH = 5.7; volume = 300 mL) with (**A**) microfiltration membrane PVDF650-PMAA, (**B**) PMAA–microglass fiber, and (**C**) PMAA–microglass composite. Error bars are shown for triplicate measurements.

Many studies have examined how pH affects the responsiveness of PMAA-functionalized membranes [47,48]. Figure 8A–C shows that water permeability varies with pH for both membrane/fiber media because PMAA hydrogel expands or shrinks according to changes in protonation/deprotonation state due to its weak acidic nature (pKa = 4.6–4.8). When PMAA is exposed to alkaline pH, it swells and in turn reduces the effective pore size of the PVDF/fiber domain, lowering water permeability as a result. Polymer chains collapse when acidic conditions exist, which produces an increase in permeability and restores pore size. After PMMA functionalization, membranes and fibers exhibited expected pH-responsive behavior. There is, however, a unique permeability dynamics for the three functionalized materials that depend on PMAA uptake capacity, intrinsic surface chemistry, and pore orientation. With limited hydrogel loading and a more compact pore network, PMAA-PVDF650 membranes show a gradual drop in permeability as pH increases, due to carboxyl group ionization and swelling. Interestingly, PMAA–microglass filters with Si-O and COOH-rich surfaces show a noticeable drop (89%) in permeability between pH 2 and 5.7 from 52 to 5.6 LMH/bar (Figure 8B). On the other hand, PMAA–microglass composite fibers with abundant PET (Polyethylene terephthalate) backing and thicker layer—which absorb highest PMAA based on weight measurements and have a looser fibrous matrix as a result—show an almost linear decrease from 169 to 120 LMH/bar (29%) (Figure 8C) over the same pH range, with an evenly distributed polymer swelling proportionate with pH, causing flow channels to gradually narrow. While microglass composite fiber is thicker and has a higher PMAA loading, it has a lower drop in flux than microglass without polyester backing, possibly due to the fact that there is less PMAA domain accessible to feed solution within the pores rather than the surface for successful ionization to alter pore size.

Water contact angle measurements at pH 5.7 were performed on both the bare and PMAA-functionalized PVDFs to evaluate changes in surface hydrophilicity after pore modification. The functionalized membrane showed a significant reduction in contact angle from 68.79° (±0.49°) to 30.84° (±1.89°), indicating enhanced water affinity following PMAA integration in Figure S4. For both functionalized and bare membranes, a sessile drop method was used in this study. The high surface free energy on highly hydrophilic surfaces makes it difficult to determine accurate contact angles using this method, and the PMAA hydrogel layers quickly absorb water, making it difficult for droplets to remain stable during measurement because of the rapid spread of the water droplet [49]. For functionalized samples, five studies were included on average, or a better method could be the captive bubble method used by our group in previous projects [29].

### 3.2. Solution Phase Nanoparticles

**Figure 9:**
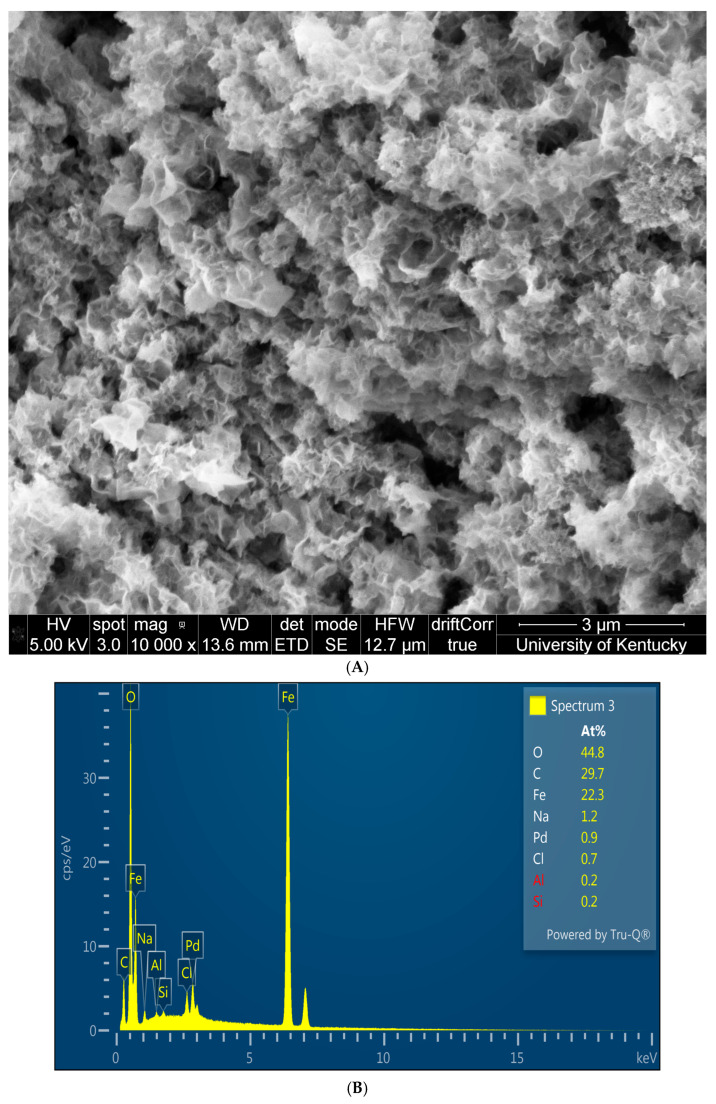

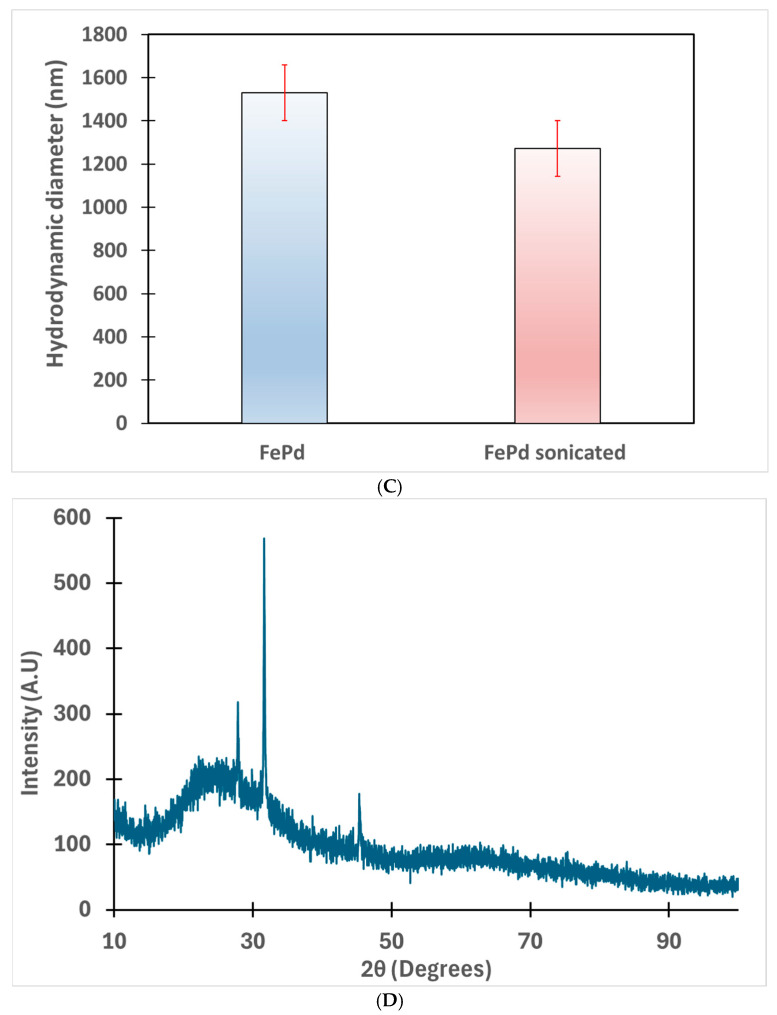
Material characterization of solution phase Fe/Pd; (**A**) SEM of fresh Fe/Pd nanoparticles; (**B**) atomic%-based EDX; (**C**) DLS analysis of fresh unsonicated and sonicated Fe/Pd; (**D**) XRD of solution phase Fe/Pd.

Catalytic Fe-Pd nanoparticles synthesized in solutions have higher tendency of agglomeration as shown by the white flakes in Figure 9A. Due to the presence of oxygen dissolved in water and in the air, the surface of Fe can be oxidized and reduced in charge. EDX analysis shows that Pd has been successfully loaded on Fe as shown in Figure 9B, along with high oxygen content along with negligible Al and Si mainly from background. Using DLS at pH 5.7, particles with a hydrodynamic diameter of 1530 nm reduced by 17% when placed in a sonicator for 10 min with a hydrodynamic diameter of 1272 nm (Figure 9C). The size of the primary particles remains constant while loose agglomerates are dispersed. Since the particles remain micro-sized, substantial clustering persists as shown in Figure S5. Fe/FexOy exhibits a strong positive charge in the acidic pH range of 2–6, and significant agglomeration occurs at higher pH, probably due to the addition of iron hydrolysis on the tough oxide barrier coupled with variation in intermolecular forces on the surface, affecting its pore size distribution [50,51]. Moreover, a multiphase Fe/Pd nanocrystalline system consistent with our previous publications can be demonstrated by peak broadening of the XRD pattern (Figure 9D) associating FCC-Pd^0^, BCC-Fe^0^, and an Fe–Pd alloy phase [29,45].

### 3.3. Fe/Pd In Situ Nanoparticles Within Microfiltration Membrane Domain

**Figure 10:**
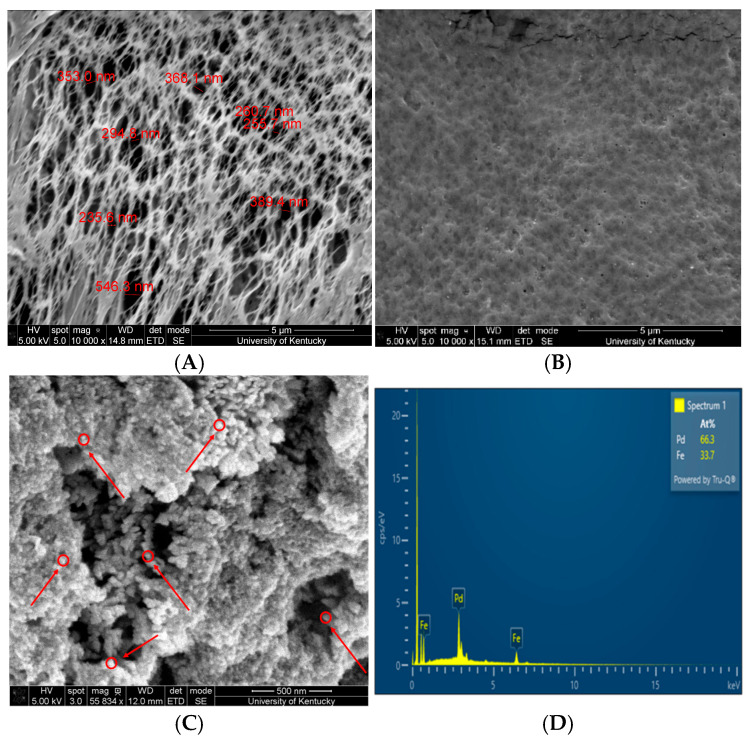
Fe/Pd in situ nanoparticles within microfiltration membrane domain: (**A**) bare PVDF650 membrane top view; (**B**) PMAA-PVDF 650 membrane top view; (**C**) Fe/Pd PMAA-PVDF 650 membrane top with arrow-pointed Fe/Pd moieties; (**D**) EDX point scan Fe/Pd PMAA-PVDF 650.

By immobilizing iron-based nanoparticles in hydrogel–membrane matrices, it is possible to prevent persistent agglomeration observed in solution phase. Figure 10A shows the platinum-coated bare PVDF-650 membrane with a pore diameter of 325 ± 101 nm. The pore size effectively reduced post-hydrogel functionalization with 15 wt% MAA monomer and 1 mol% APS/MBA due to significant PMAA chain formation as shown in Figure 10B, exhibiting similar membrane parameters found by our group’s earlier studies by Wan et al., 2020 [45]. As a result of embedding particles directly into the compact polymeric domain, their mobility is limited, surface oxidation is reduced, and catalytic efficiency is enhanced since the high density of reactive sites are available for degrading priority contaminants. Clusters of Fe/Pd of size between 43.0 and 64.3 nm (Figure 10C) within the PMAA-PVDF matrix span over the surface and across the cross section using the chemical synthesis approach used in this paper. Nevertheless, it is worth mentioning that using various crosslinking densities, Fe/Pd nanoparticles can be made smaller by reducing their mobility and overcoming mass transfer constraints. EDX point scan further proves the existence of Fe/Pd moieties within the fabricated sample with Fe/Pd at% of approximately 2.0 (Figure 10D); that just gives composition in the point/mapping area, rather than finding out the exact metal-loading capacity in the entire sample which is the limitation of the technique [41]. ICP-OES characterization of the Fe/Pd-loaded poly(acrylic acid)–functionalized PVDF (Fe/Pd PAA-PVDF) with an effective area of 13.2 cm^2^ indicated a nanoparticle (16–19 nm)-loading density of 0.41 g/L and acid digestion of the sample in 20% HNO_3_ revealed that Pd accounted for 11.2 wt% relative to Fe with negligible leaching due to ion exchange chemistry proving long-term stability [41]. Using the same convective loading approach as described in Section 2.3, another study by our group found a Pd-to-Fe ratio of approximately 12 wt% on PMAA-PVDF, with less than 2 wt% metal loss after filtering 179 L/m^2^ of water at 1 bar with the PMAA hydrogel network effectively retaining embedded nanoparticles (17 nm) and minimizing leaching [45]. In addition, Fe/Pd PMAA-PVDF650 membranes prepared through the convective ion-exchange method and stored in ethanol at 4 °C remained catalytically active for over 21 days. Within this period, the membranes were successfully employed for the hydrogenation of 4-chlorophenol to phenol in the present study.

### 3.4. Halo-Organic Degradation Studies in Batch and Convective Flow Mode

Extensive work has examined Fe/Pd-based catalytic systems in which hydrogen is supplied externally, produced in situ through iron corrosion, or generated biologically by phototrophic bacteria. Our group has evaluated these pathways extensively for the reductive transformation of chlorinated organics under ambient conditions, using both batch reactors and convective flow membrane systems. One line of study focused on iron and iron oxide nanoparticles (20–50 nm) encapsulated in sulfonated silica. These particles were synthesized by reducing ferric ions immobilized through ion exchange and were subsequently used for degrading trichloroethylene (TCE) through both oxidative and reductive mechanisms. In oxidative operation, air-exposed iron/silica nanoparticles partially oxidized to form reactive iron oxide surfaces capable of decomposing hydrogen peroxide into short-lived hydroxyl radicals. For a 20 mL solution of TCE (21.5 mg/L) containing 0.16 g/L Fe, roughly 50% dechlorination was achieved at pH 7 within 50 h, corresponding to a pseudo-first-order rate constant (kobs) of 0.034 h^−1^ [45]. Under reducing conditions, hydrogen generated during Fe^0^ corrosion produced reactive hydrogen radicals on Pd surfaces, enabling hydrodechlorination of TCE via classical pathways:

Fe^0^ + 2H_2_O → Fe^2+^ + H_2_ + 2OH^−^ (2)

H_2_ + Pd^0^ → Pd-H intermediate complex → 2H* + Pd^0^ (3)

H* + R-X (halogenated organics) → R-H (hydrogenated products) + X^−^ (Halide) (4)

Using 1 g/L Fe to treat water containing 27 mg/L TCE, this approach yielded 37% dechlorination in 8 h, with a surface-area-normalized rate constant (ksa) of 8.1 × 10^−4^ L/m^2^·h and kobs of 0.06 h^−1^. While zero-valent iron can convert TCE to ethylene through electron-transfer reactions, Pd-coated iron significantly accelerates reaction kinetics by catalyzing hydrogen formation and promoting spontaneous hydrogenation [52]. To extend these concepts to membrane systems, our group developed a method to metallize ultrafiltration membranes through magnetron sputtering. A 10 nm tantalum coating reduced pore size from 19 to 6 nm and decreased water permeability from 168 LMH/bar to 8.8 LMH/bar. A catalytic Mg/Pd nanoporous layer was also integrated; dealloying of Mg in water generated Pd ligaments roughly 4 nm in size. These membranes were evaluated in convective flow hydrogenation of TCE and PCB-1. At 4 bar, water containing 6.2 mg/L dissolved hydrogen achieved 58% TCE reduction with a 4.8 ms residence time. At 8 bar, although dissolved hydrogen increased to 12.3 mg/L, only 45% reduction occurred due to the shorter residence time of 2.8 ms. Most dechlorination took place in the retentate, implying that reaction kinetics were governed by both hydrogen radical availability and contact time between contaminants and catalytic sites. Increased biphenyl yield at higher dissolved hydrogen concentrations further highlighted the importance of hydrogen availability [53]. Earlier work from the group demonstrated alternative hydrogen generation through phototrophic bacteria [54,55]. Waste organic acids (80 mM) supplied to *Rhodopseudomonas palustris* produced hydrogen as a byproduct of nitrogen fixation, and the process was enhanced using 120 nm silica particles embedded with 16 nm gold nanoparticles activated by near-infrared (NIR) light. In 40 mL batch cultures, NIR illumination increased hydrogen output from 60 μmol to 167 μmol—more than a 2.5-fold improvement [54]. Biohydrogen generated this way was integrated with Fe/Pd catalysts to degrade PCB-type chloro-organics. More than 90% of PCB-1 was converted to biphenyl within 5 h using either 1% Pd/Al_2_O_3_ or Pd-functionalized PVDF (0.041 mg/cm^2^ Pd loading). A total of 0.135 mmol H_2_ was generated—approximately 3.5 times the theoretical requirement for complete PCB-1 hydrogenation—and supplied to catalytic reactors operating at room temperature. Pd/Al_2_O_3_ achieved faster degradation (40.2 h^−1^·mg^−1^) than Pd-coated membranes (11.5 h^−1^·mg^−1^) due to improved particle mobility and mass transfer, while membrane-bound reactions were limited by hydrogen diffusion. Consequently, hydrogen partial pressure emerged as a critical determinant of hydrogenation efficiency in membrane-based catalytic systems [55].

**Figure 11:**
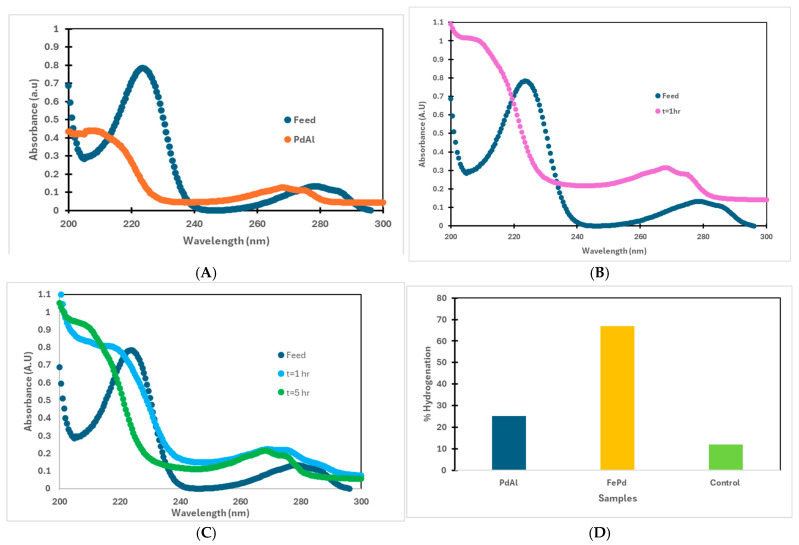
Batch hydrogenation of 4-Chlorophenol and PFOA all at 22–23 °C with hydrogen bubbled at 0.14 bar at pH 5.7; (**A**) 10 mg/L 4-CP solution was hydrogenated for 4 h with 0.2 mg/L commercial Pd-on-alumina (1 wt% Pd loading) particles; (**B**) 10 mg/L 4-CP hydrogenated for 1 h with 0.25 mg/L freshly synthesized solution phase sonicated Fe/Pd bimetallic nanoparticles; (**C**) 400 mL of 10 mg/L 4-CP treated for 5 h with chopped in situ Fe/Pd PMAA PVDF-650; (**D**) 1 mg/L PFOA hydrogenated with 0.2 mg/L Pd on alumina, 0.25 mg/L solution phase Fe/Pd, and a control run with 0.25 mg/L Fe/Pd, where ultrapure hydrogen was replaced with nitrogen under the same setup.

However, in this study, using a continuous ultrapure hydrogen gas supply as shown in Figure S6A–D, 10 mg/L 4-CP solution was treated at 22–23 °C for 4 h at pH 5.6 and with 0.2 mg/L commercial Pd-on-alumina particles. A reductive dechlorination peak revealed in the UV-vis spectrum (Figure 11A) indicates that 4-CP has been completely converted to phenol, confirming effective hydrogen supply. To further verify the efficiency of the batch reaction, 10 mg/L 4-CP was degraded under the same thermal and pH conditions using 0.25 mg/L of freshly synthesized Fe/Pd bimetallic nanoparticles. After an hour, phenol was again produced as a result of the degradation process (Figure 11B). The absence of transformation was observed when nitrogen gas was substituted for hydrogen, demonstrating that molecular hydrogen is a crucial reductant and source of reactive hydrogen species, adsorbing onto Pd surfaces that mediate hydrodechlorination via electron-rich intermediates. A batch mode treatment of 400 mL of 10 mg/L 4-CP at pH 5.7 and ambient temperature was performed using Pd-Fe nanoparticles immobilized on PMAA-functionalized PVDF-650 membranes (surface area 14.6 cm^2^). A complete conversion to phenol was again observed over a 1–5 h reaction period, proving the catalytic membrane’s efficiency and stable immobilization of active sites within the polymer matrix (Figure 11C). Finaly, the process was modified for convective flow conditions using the same Fe/Pd PMAA-PVDF650 membrane for treating 200 mL of 10 mg/L 4-CP at pH 5.7 with residence times between 1 and 20 min. In spite of a measurable decrease in 4-CP concentration, the transformation was incomplete, likely due to the limited residence time that prevented effective contact between 4-CP, the catalytic Pd sites on membrane, and hydrogen radicals adsorbed on the Pd sites. It suggests that if continuous treatment systems are to be effective, flow rate, and membrane design should be optimized to ensure adequate fluid–catalyst interaction and residence time.

This work represents the evaluation of the hydrogenation efficiency of perfluoro-organics (PFOA) using solution-phase Fe/Pd nanoparticles prepared via the synthesis route described in Section 2.4. Targeted analysis confirmed a significant reduction in PFOA concentration in the presence of the catalyst. While commercial Pd on alumina achieved only 25% conversion, the lab-synthesized Fe/Pd nanoparticles delivered a markedly higher 67% hydrogenation under identical conditions (Figure 11D). Experiments were conducted for 3 h using 0.25 mg/L of sonicated solution phase Fe/Pd nanoparticles at 20–22 °C and with feed pH 5.7 and 1 mg/L PFOA is diluted to <100 ppb before analysis. A control experiment, where hydrogen was replaced with nitrogen under the same setup, showed only a 12% decrease, underscoring the role of Fe/Pd surface chemisorption in forming a strong PFOA–Fe/Pd complex, as also discussed by Long et al. (2021) [56].

## 4. Conclusions

Our work has advanced the reactive membrane field by quantified demonstration of the synthesis of responsive membranes with the metal catalyst domain and in situ reductive hydrogenation of selected halo-organics. Poly (methacrylic acid), a relatively low-volatility monomer, showed successful functionalization of microfiltration membranes and fiber filter domains. Post-functionalization weight-gain analysis indicates that PVDF-650 exhibits a 25% increase in mass, while microglass fiber and microglass composite show substantially higher gains of 116% and 137%, respectively. Correspondingly, the pure-water flux of PVDF-650 declines from 509 to 148 LMH/bar, microglass fiber drops sharply from 3392 LMH/bar to 5.6 LMH/bar, and microglass composite decreases from 5144 LMH/bar to 120 LMH/bar. The PMAA-modified PVDF-650 membrane was further developed into a catalytic Fe/Pd PMAA-PVDF-650 system, displaying a flux of 20.7 LMH/bar and an Fe/Pd atomic percentage of roughly 2.0%, as confirmed by point-scan EDX. The embedded nanoparticles fall within the 43–64 nm size range. Additionally, Fe/Pd nanoparticles synthesized in solution show extensive agglomeration, forming clusters between 1272 and 1530 nm, and XRD analysis indicates alloy-type crystalline structures. A total of 0.25 mg/L Fe/Pd nanoparticles synthesized in solution demonstrated a strong catalytic performance, achieving complete hydrogenation of 4-chlorophenol to phenol and 67% hydrogenation of PFOA to its reduced form at 22–23 °C, with the ultrapure hydrogen gas supply at pH 5.7. Collectively, these findings point to a versatile and scalable strategy for pollutant degradation at a lower temperature, establishing hybrid catalytic–membrane systems as a promising platform for advancing water treatment technologies with our future work involving development of catalytic Fe/Pd polymeric fiber media for advanced water treatment.

## Figures and Tables

**Table 1:** Microfiltration platform used for this study.

Microfiltration Media	Functionalization	Catalyst Incorporation
1. Microfiltration membrane PVDF 650	15 wt% PMAA and 1 mol% APS + 1 mol% MBA used for functionalization to synthesize: (a) PMAA-PVDF650	Fe/Pd nanoparticles are incorporated only in PMAA-PVDF650 membrane to synthesize *Fe/Pd PMAA-PVDF650* membrane illustrated in Figure 3.
2. Polymeric non-woven fiber filters (a) Bare microglass w/o polyester backing (microglass) (b) Microglass w/polyester backing (microglass composite)	15 wt% PMAA and 1 mol% APS + 1 mol% MBA used for functionalization to synthesize: (a) PMAA–Microglass (b) PMAA–Microglass composite	The fiber filters exhibit pH-responsive water permeability; however, no catalyst is incorporated, as all hydrogenation experiments are conducted exclusively using PVDF.

## Data Availability

The raw research data supporting the conclusions of this article will be made available by the authors on request.

## Supplementary Materials

The following supporting information can be downloaded at https://www.mdpi.com/article/10.3390/nano16010014/s1; Figure S1: Hydrogel appearance with 15 wt% MAA, 1 mol% APS and 1–2 mol% MBA polymerized in vacuum oven for 2 h at 85 °C; Figure S2: SEM images of unmodified polymeric non-woven fiber filters used for PMAA functionalization; (A) top view of microglass composite with PE backing (microglass fiber length 14.4 ± 1.3 µm); (B) rear view of microglass composite with PE backing (PET fiber length 17.2 ± 1.6 µm); (C) cross-sectional view of microglass composite with PE backing (thickness 830 µm); (D) top view of pristine microglass without PE backing (microglass fiber length 15.8 ± 1.7 µm); (E) rear view of pristine microglass without PE backing; (F) cross-sectional view of pristine microglass without PE backing (thickness 647 µm); Figure S3: Pure water flux of bare PVDF microfiltration membranes, PMAA-functionalized membranes, and Fe/Pd nanoparticle-loaded PMAA–PVDF, evaluated under ambient laboratory conditions at pH 5.7 and 22 °C to assess the impact of functionalization and catalytic nanoparticle incorporation on intrinsic permeability; Figure S4: Contact angle decay of bare and functionalized membrane with using 10 µL droplets of deionized water at pH 5.7 on a Krüss DSA100S drop shape analyzer; Figure S5: Dynamic light scattering analysis of solution-phase Fe/Pd nanoparticles, comparing sonicated and unsonicated samples (3 mL each) placed in quartz or disposable cuvettes, with precautions taken to prevent air bubble formation and cuvette surface contamination (cleaned using Kimwipes); measurements performed in triplicate at 22 °C; Figure S6: Representative images of the functionalized catalytic membrane system and experimental setup; (A) appearance of functionalized and nanoparticle-loaded membrane; (B) solution phase Fe/Pd nanoparticles; (C) membrane cell for flux studies; (D) temperature controlled batch hydrogenation setup.

## Abbreviations

The following abbreviations are used in this manuscript:

PFAS	Per/Polyfluoroalkyl substances
PFOA	Perfluorooctanoic acid
PFOS	Perfluorooctane sulfonic acid
PMAA	Poly methacrylic acid
PVDF	Polyvinylidene fluoride
PE	Polyester
PET	Polyethylene terephthalate
XRD	X-ray diffraction
SEM	Scanning electron microscopy
EDX	Energy dispersive spectroscopy
FT-IR	Fourier Transform Infrared Spectroscopy
TCE	Trichloroethylene
PCB	Polychlorinated biphenyl
