# Supplementary material for: Functionalization of Microfiltration Media Towards Catalytic Hydrogenation of Selected Halo-Organics from Water
Source: Nanomaterials (Basel). 2025 Dec 22;16(1):14. doi: 10.3390/nano16010014 (PMC12787646; doi:10.3390/nano16010014)
Supplement: Supplementary file 1 [file nanomaterials-16-00014-s001.zip › nanomaterials-4053515-supplementary.pdf]

Supporting information

Functionalization of Microfiltration media towards catalytic hydrogenation of selected halo-organics from water

Subrajit Bosu [a], Samuel. S. Thompson [b], Doo Young Kim [c], Noah D. Meeks [d] and Dibakar Bhattacharyya [a*]

[aba*]Department of Chemical and Materials Engineering, University of Kentucky, Lexington, KY 40506, USA
[c]Department of Chemistry, University of Kentucky, Lexington, Kentucky, United States
[d]Southern Company Services, Inc., Birmingham, AL 35203, USA.

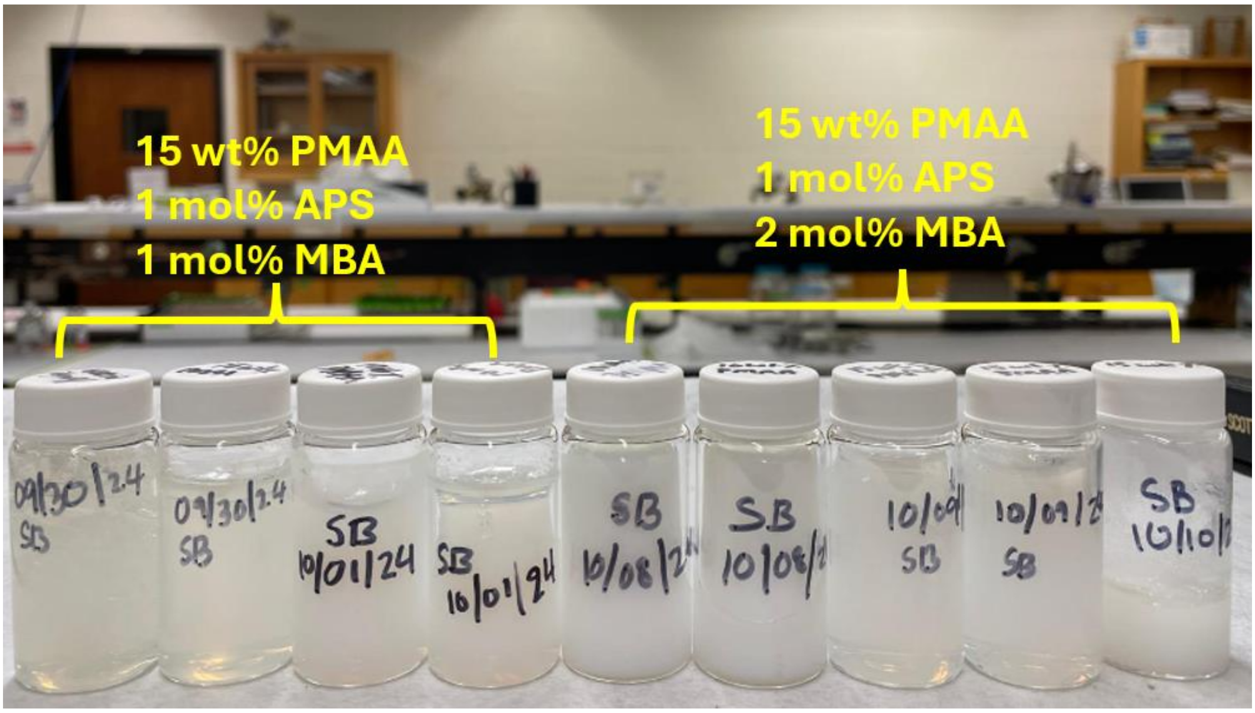

Figure S1. Hydrogel appearance with 15 wt% MAA, 1 mol% APS and 1-2 mol% MBA polymerized in vacuum oven for 2 hours at 85 °C.

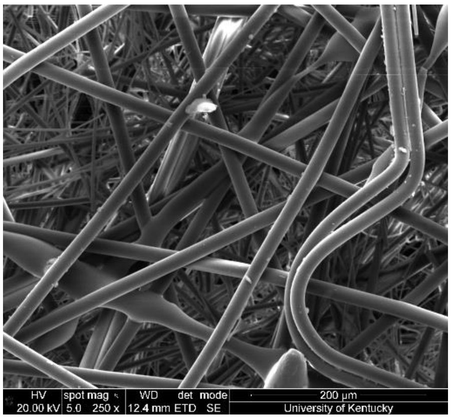

(A)

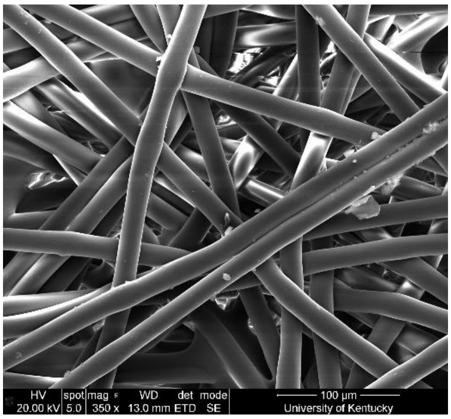

(B)

.

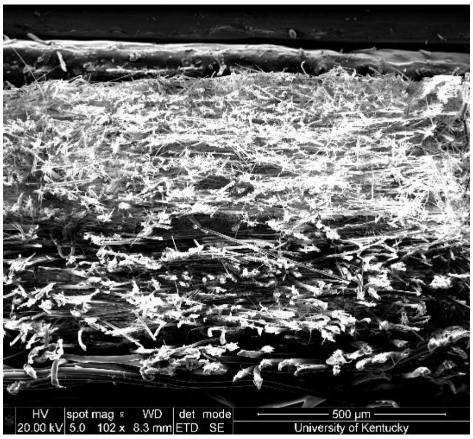

(C)

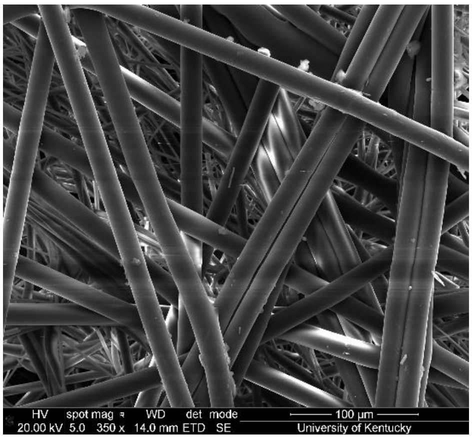

(D)

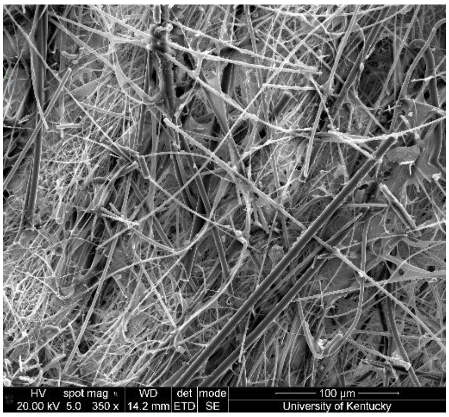

(E)

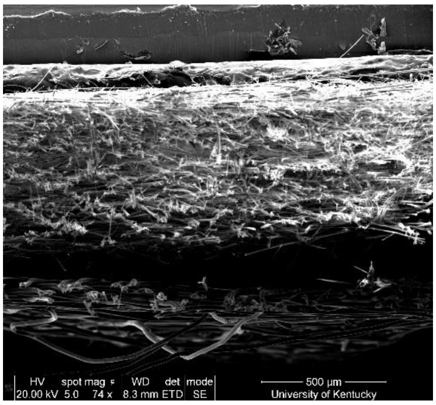

(F)

Figure S2. SEM images of unmodified polymeric non-woven fiber filters used for PMAA functionalization **(A)** top view of micro glass composite with polyester backing (micro glass fiber length 14.4 ± 1.3 μm) (**(B)** rear view of micro glass composite with polyester backing (PET fiber length 17.2 ± 1.6 μm) **(C)** cross sectional view of micro glass composite with backing (thickness 830 μm) **(D)** top view of pristine micro glass without polyester backing (micro glass fiber length 15.8 ± 1.7 μm) **(E)** rear view of pristine micro glass without polyester backing **(F)** cross sectional view of pristine micro glass without polyester backing (thickness 647 μm).

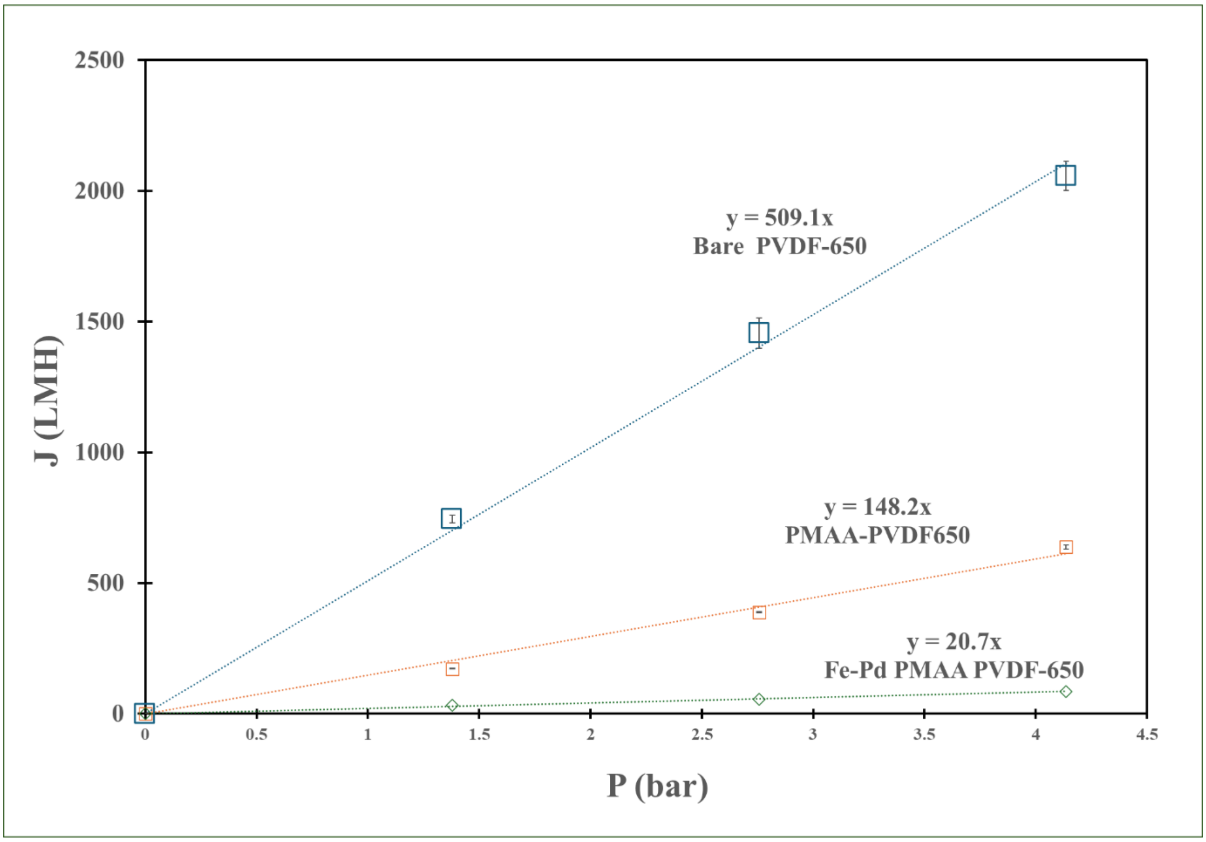

Figure S3. Pure water flux of bare PVDF microfiltration membranes, PMAA-functionalized membranes, and Fe/Pd nanoparticle-loaded PMAA–PVDF membranes, evaluated under ambient laboratory conditions at pH 5.7 and 22-23 °C to assess the impact of functionalization and catalytic nanoparticle incorporation on intrinsic pure water permeability. Error bars are shown for triplicate measurements.

Water flux is directly related to the natural permeance of the membrane and the applied pressure gradient in the first component of the equation. The following component of the equation (see Equation 1) enables a theoretical calculation of the equivalent pore radius (Rp), assuming that key parameters such as membrane thickness (L), water viscosity (μ), and porosity (ε)—typically taken as 0.5 for porous structures—are known. Using these relationships, the water permeance and pore size characteristics of synthesized membrane systems will be evaluated. In situ polymerization of carboxyl-based monomers within the membrane structure will reduce the effective pore size while significantly decreasing water permeance shown above.

$$J_W = A_M \Delta P = \frac{\varepsilon R_p^2}{8 \mu L} \Delta P \quad \text{(S1)}$$

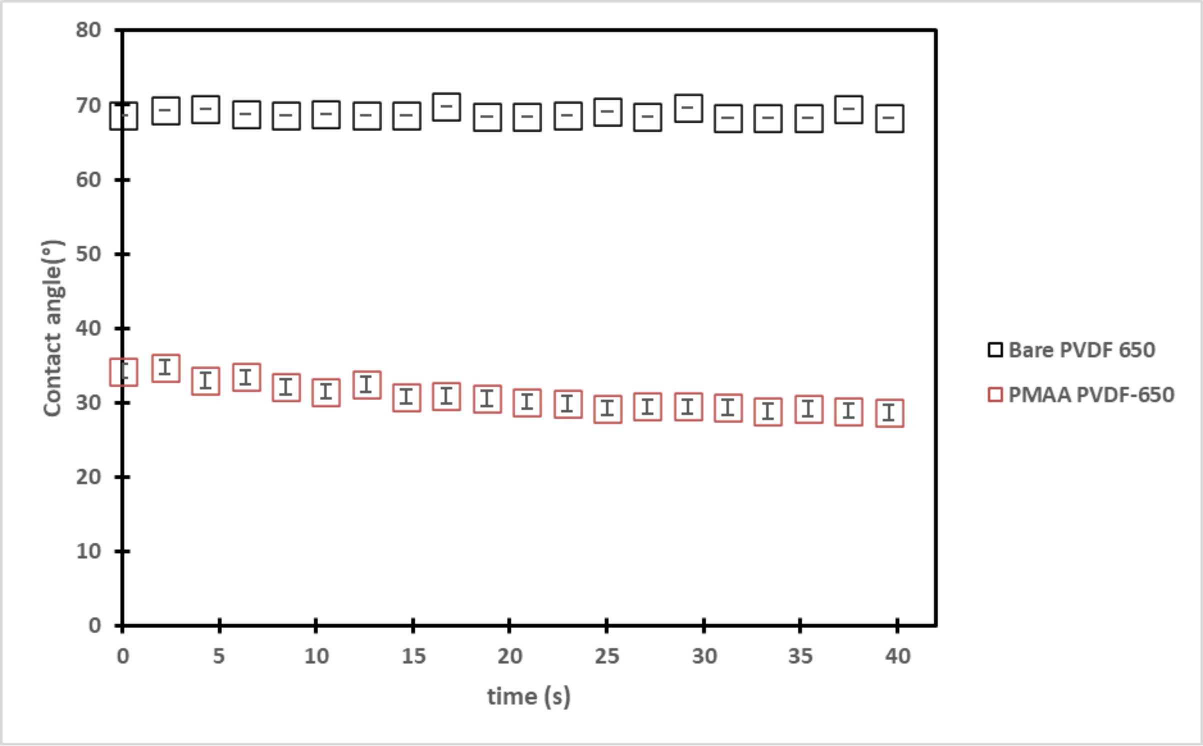

Figure S4. Contact angle decay of bare and functionalized membrane with using 10 μL droplets of deionized water at pH 5.7 on a Krüss DSA100S drop shape analyzer. Error bars are shown for triplicate measurements.

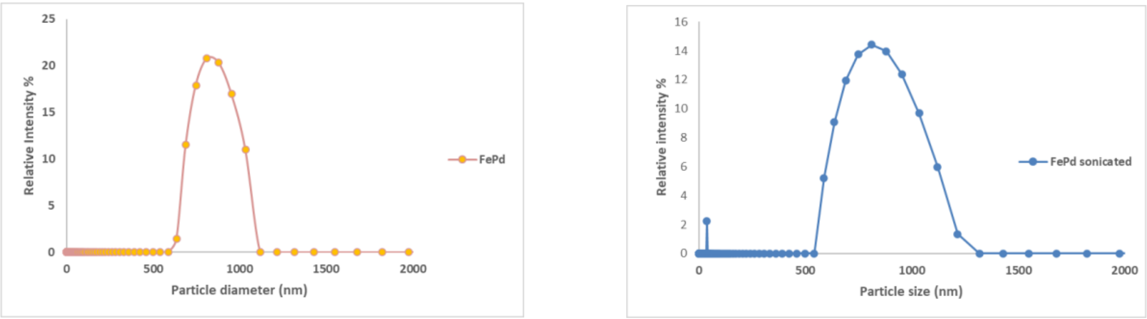

(A) **Fe/Pd unsonicated** **(B)** **Fe/Pd sonicated**

Figure S5. Dynamic light scattering analysis of solution-phase Fe/Pd nanoparticles, comparing sonicated and unsonicated samples (3 mL each) placed in quartz or disposable cuvettes, with precautions taken to prevent air bubble formation and cuvette surface contamination (cleaned using Kimwipes); measurements performed in triplicate at 22 °C.

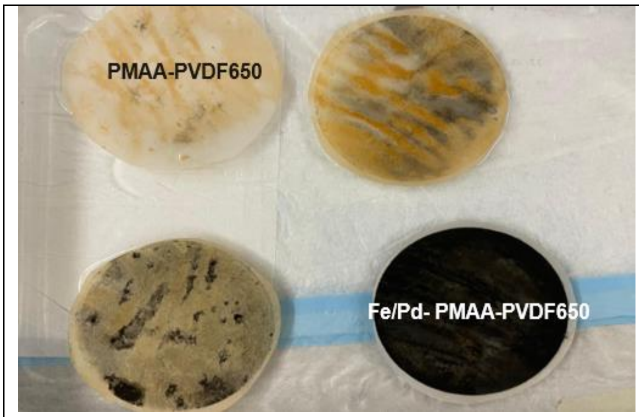

(A)

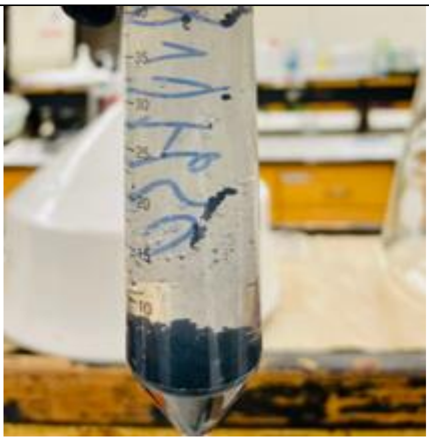

(B)

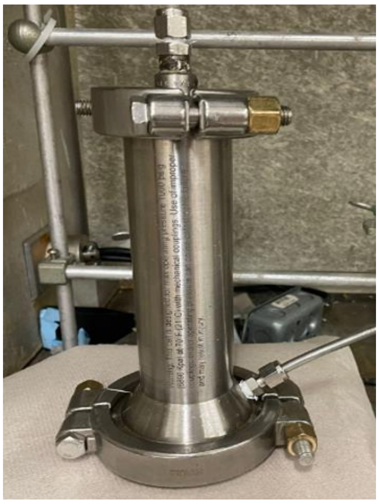

(C)

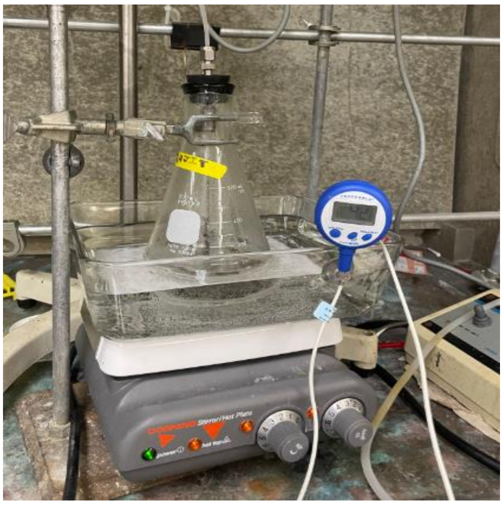

(D)

Figure S6. Representative images of the functionalized catalytic membrane system and experimental setup **(A)** Appearance of functionalized and Fe/Pd nanoparticle loaded membrane **(B)** Solution phase Fe/Pd nanoparticles **(C)** Membrane cell for flux studies **(D)** Temperature controlled batch hydrogenation setup.
